# Combined coagulation and inflammation markers as predictors of venous thrombo-embolism and death in COVID-19

**DOI:** 10.3389/fmed.2024.1399335

**Published:** 2024-06-10

**Authors:** Jaja Zhu, Raïda Bouzid, Benoît Travert, Guillaume Géri, Yves Cohen, Adrien Picod, Nicholas Heming, Martin Rottman, Bérangère Joly-Laffargue, Agnès Veyradier, Claude Capron, Paul Coppo

**Affiliations:** ^1^Service d’Hématologie-Immunologie-Transfusion, AP-HP Paris Saclay, CHU Ambroise Paré, Université de Versailles Saint Quentin-Université Paris Saclay, Montigny-le-Bretonneux, France; ^2^Laboratoire Cellules Souches et Applications Thérapeutiques, UMR INSERM 1184, Commissariat à l’Energie Atomique et aux Energies Alternatives, Fontenay-aux-Roses, France; ^3^Centre de Référence des Microangiopathies Thrombotiques (CNR-MAT), AP-HP, Paris, France; ^4^Service de Médecine Interne, Hôpital Ambroise-Paré, AP-HP, Boulogne-Billancourt, France; ^5^Service de Médecine Intensive et Réanimation, Hôpital Ambroise-Paré, AP-HP, Boulogne-Billancourt, France; ^6^Service de Médecine Intensive et Réanimation, Hôpital Avicenne, AP-HP, Paris, France; ^7^Department of Intensive Care, Raymond Poincaré Hospital, APHP University Versailles Saint Quentin-University Paris Saclay, Garches, France; ^8^Institut Hospitalo Universitaire PROMETHEUS, Garches, France; ^9^Innovative Biomarkers Plateform, Laboratory of Infection & Inflammation-U1173, School of Medicine, INSERM, University Versailles Saint Quentin-University Paris Saclay, Garches, France; ^10^FHU SEPSIS, Garches, France; ^11^Innovative Biomarkers Plateform, Laboratory of Infection & Inflammation-U1173, School of Medicine, INSERM, University Versailles Saint Quentin-University Paris Saclay, Garches, France; FHU SEPSIS, Garches, France; ^12^General Intensive Care Unit, Raymond Poincaré Hospital (AP-HP), FHU SEPSIS, Laboratory of Infection and Inflammation-U1173, School of Medicine Simone Veil, Université Versailles Saint Quentin, University Paris Saclay, INSERM, Garches, France; ^13^EA3518, Institut de Recherche Saint Louis, Université de Paris, Paris, France; ^14^Service D’hématologie Biologique, Laboratoire ADAMTS13, Hôpital Lariboisière, AP-HP Nord, Université de Paris, Paris, France; ^15^Université Paris-Saclay, Université de Versailles Saint Quentin en Yvelines (UVSQ), Biomarqueurs en cancérologie et onco-hématologie (BECCOH), Boulogne-Billancourt, France; ^16^Service d’Hématologie, Hôpital Saint-Antoine, AP-HP-Sorbonne Université, Paris, France; ^17^NSERM UMRS 1138, Centre de Recherche des Cordeliers, Paris, France

**Keywords:** thrombosis, COVID-19, ADAMTS13, von Willebrand factor, interleukin-6, C-reactive protein, prognosis

## Abstract

**Background:**

The COVID-19 pandemic related to SARS-CoV-2 virus was responsible for global pandemic. The severe form of the disease was linked to excessive activation of immune pathways together with a systemic cytokine storm response and thrombotic venous or arterial complications. Factors predicting severe outcomes including venous and/or pulmonary thrombosis (VT) and death were identified, but the prognostic role of their combination was not addressed extensively.

**Objectives:**

We investigated the role of prognostic factors from the coagulation or inflammatory pathways to better understand the outcome of the disease.

**Methods:**

For this, we prospectively studied 167 SARS-CoV-2-positive patients from admission in intensive care units (ICU) or emergency departments from four academic hospitals over a 14-month period. Besides standard biology, we assessed serum concentrations of inflammatory markers, coagulation factors and peripheral blood cells immunophenotyping.

**Results:**

Thirty-nine patients (23.3%) developed VT and 30 patients (18%) died. By univariate analysis, C-reactive protein (CRP) level > 150 mg/L, interleukin-6 (IL-6) ≥ 20 pg/mL, D-dimers > 1,500 μg/L, ADAMTS13 activity ≤ 50%, Von

**Conclusion:**

A combination of coagulation and inflammatory markers can refine the prognostication of severe outcome in COVID-19, and could be useful for the initial evaluation of other types of viral infection.

## Graphical Abstract

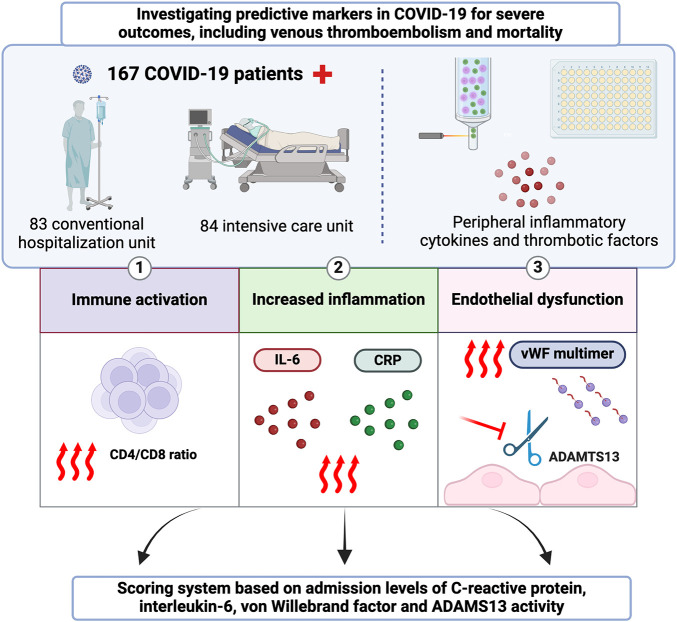

## Introduction

The severe acute respiratory syndrome (SARS) disease caused by the human SARS coronavirus 2 (SARS-CoV-2) has been responsible for the COVID-19 pandemic (1). The major advances in the understanding of the natural history and pathophysiology of the disease helped better clarifying its prognosis, now tampered by vaccination. This may apply in the future to other viral infections and could prove useful to explore and better manage infections by new SARS variants.

COVID-19 patients were mostly asymptomatic or mildly symptomatic. However, 5% of them developed a critical form of the disease with severe pulmonary damages with hypoxemia (2) The profound inflammatory response linked to an excessive activation of immune pathways, together with the related systemic cytokine storm response, led for these patients to an acute respiratory distress syndrome that induced thrombosis in pulmonary vessels (3), increasing mortality (4).

The coagulation system is activated in response to infection by a variety of different pathogens, including bacteria and viruses. This response appears to have developed as a host defense system to limit the spread of the pathogen. During infections, there is an interplay between blood coagulation, immune cells, and platelets to restrict dissemination of pathogens within the body. The activation of coagulation is beneficial for infections with bacteria and viruses by limiting pathogen dissemination and supports pathogen killing and tissue repair. On the other hand, over-activation can lead to thrombosis with subsequent depletion of hemostatic factors and secondary bleeding (5). This scenario applies for COVID-19 (6); in that regard, venous and/or pulmonary thrombosis (VT) in patients with COVID-19 has been shown to involve multiple mechanisms, including activation of coagulation pathways, endothelial cells dysfunction, release of neutrophil extracellular traps (NETs), systemic inflammation and activation of the complement system (7). Consistent with endothelial cell activation, it has been proposed that abnormal, Von Willebrand factor (VWF)-mediated, interactions between platelets and the endothelium might also contribute to thrombosis in severe forms of COVID-19 (8–11). In this regard, it has been suggested that the VWF-cleaving protease ADAMTS13/VWF antigen ratio could be related to disease severity and predict poor outcome when elevated (12–14). Reflecting the coagulopathy of severe COVID-19, abnormal coagulation parameters including increased D-dimers and fibrinogen levels, mildly prolonged prothrombin time (PT) and mild thrombocytopenia have also been associated with disease severity and poor prognosis (15–18). A role for tissue factor (TF) (factor III, tissue thromboplastin or CD142) was also suggested in COVID-19-related thrombosis (7, 19). Lastly, the acute inflammatory response to SARS-CoV-2 has been shown to induce multiorgan failure, through the production of pro-inflammatory cytokines, notably interleukin (IL)-1β and IL-6 (20). In patients with severe COVID-19, a relationship has been reported between the extent of endothelial dysfunction and the magnitude of the immune inflammatory response (20). Especially, a unified pathophysiological hypothesis suggests that the imbalance between angiotensin-II and angiopoietin_1,7_, caused by the interaction between SARS-CoV-2 and the angiotensin converting enzyme 2 (ACE2), results in an angiotensin-II “intoxication” with an abnormal activation of the angiotensin-II/angiotensin-II type 1 receptor, producing end-organ damage through the production of inflammatory cytokines and activation of the coagulation and complement cascades (21). In this way, several biomarkers of a pathway involving angiotensin-II, cytokines, C-reactive protein (CRP), coagulation (including ADAMTS13 and its substrate VWF) and finally peripheral blood immune cells have been individually described to predict the outcome of COVID-19 (22). Moreover, differences in ACE2 expression are linked to the severity and outcome of COVID-19 patients (23). Most of these markers were explored for their prognostic value; however, the role of their combination in the prognostication of severe outcomes including death, VT and ICU admission in COVID-19 patients was not fully addressed. We attempted here to assess simultaneously the role of coagulation and inflammation markers in order to better identify patients with COVID-19 at risk of severe outcomes.

## Materials and methods

### Patients

SARS-CoV-2-positive patients were prospectively and consecutively enrolled in four centers of the Assistance Publique-Hopitaux de Paris (AP-HP) (Avicenne, Ambroise Paré, Raymond-Poincaré and Lariboisière) from March 2020 to April 2021. Inclusion criteria allowed to select severe COVID-19 forms as enrolled patients had to be treated in intensive care unit (ICU) or hospitalized in emergency departments. All cases were confirmed as being positive by reverse transcription polymerase chain reaction (RT-PCR) of nasal swabs or tracheal aspirates. Clinical characteristics were collected from patient charts. VT was suspected clinically during hospitalization and systematically confirmed by Doppler-ultrasonography or computerized tomography.

Blood cell count, standard coagulation tests [prothrombin time (PT), activated partial thromboplastin time (ATT), fibrinogen and D-dimer] and serum CRP were assessed on admission.

Written informed consent was obtained from all participants or representatives. The study protocol at all sites was approved by the Ethics committee: CE SRLF 20-29 and CER-Paris-Saclay-2020-050. For all patients, the following blood sample analyses were issued on first admission as part of the routine care; therefore, no additional sampling was performed for the study.

### Flow cytometry

Peripheral blood (PB) was processed for immunophenotyping as reported (24), Briefly, PB was stained with fluorochrome-conjugated antibodies to CD45, CD3, CD4, CD8, CD14, CD16, CD38 and HLA-DR to evaluate lymphocyte subsets, monocytes and activated cells. The expression of TF on monocytes was investigated with antibodies to CD142. All antibodies were purchased from BD Biosciences (Supplementary Table 1). Samples were acquired on a BD FACS Lyric instrument (BD Biosciences) and data were further analyzed with the Kaluza software (Beckman Coulter).

### Peripheral inflammatory cytokines and thrombotic factors

IL-1β, IL-6, angiotensin-II and TF activity levels were measured using standard commercially available enzyme-linked immunosorbent assay (ELISA) kits according to manufacturer instructions (Duoset reagents from Bio-Techne; LSBIO and Abcam, respectively). ADAMTS13 activity, VWF antigen (VWF:ag) and VWF cofactor binding activity (VWF:CB) were assessed in plasma as previously described (10).

### Statistical analyses

Qualitative variables are reported as numbers and percentages. Quantitative discrete and continuous variables are reported as medians and interquartile ranges (IQR). Pearson’s Chi Square was used for comparison of qualitative variables and Mann-Whitney test to compare quantitative variables between subgroups. In order to identify factors independently associated with VT and mortality, a Cox proportional hazards regression was performed. The Youden Index was calculated to identify optimal cut-off values for the parameters found to be significant in univariate analysis (sensitivity + specificity–1). The sensitivity, specificity, positive / negative predictive values and their respective confidence intervals (CIs) were determined. The discriminative performance of a scoring system in predicting death and the likelihood of being in ICU were assessed using the area under the receiver operating characteristic (ROC) curve (AUC). Statistical analyses were performed with R version 3.6.1 (2019-07-05) (The R Project).^1^

## Results

**FIGURE 1:**
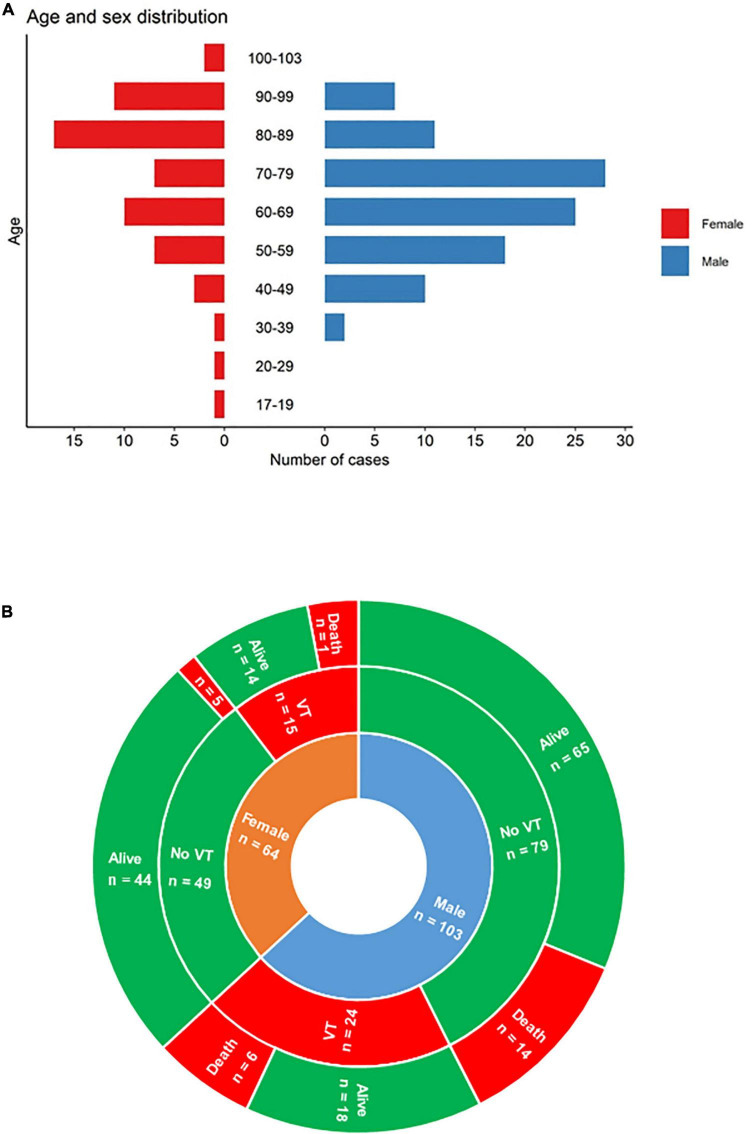
**(A)** Demographic data; **(B)** main severe outcomes (thrombo-embolic events and survival) according to sex. VT, venous or pulmonary thrombosis.

During the inclusion period, 167 COVID-19 patients were enrolled. About half of these patients (50.3%) were in ICU at the time of the study. Of the 167 patients, 39 (23.3%) developed VT and 30 (20%) died (Table 1 and Figures 1A, B).

**TABLE 1:** Patient characteristics at diagnosis.

	Total *n* = 167	No VT *n* = 128	VT *n* = 39	*p*-value	Survivors *n* = 137	Non-survivors *n* = 30	*p*-value	No ICU *n* = 83	ICU *n* = 84	*p*-value
Age (years)	72 [60–85]	73 [61–87]	71 [58–82]	0.38	71 [58–84]	74.5 [67–88]	0.11	83 [64–91]	66.5 [56–75]	**< 0.001**
**Gender**										
Female, *n* (%)	64 (38.3)	49 (38.3)	15 (38.5)	0.98	58 (42.3)	6 (20)	**< 0.001**	45 (54.2)	19 (22.6)	**0.023**
Male, *n* (%)	103 (61.7)	79 (61.7)	24 (61.5)		79 (57.7)	24 (80)		38 (45.8)	65 (77.4)	
**Comorbidity**										
Obesity, *n* (%)	43 (25.8)	37 (28.9)	6 (15.4)	0.09	34 (24.8)	9 (30)	0.56	12 (14.5)	31 (36.9)	**0.001**
Smoking, *n* (%)	37 (22.2)	26 (20.3)	11 (28.2)	0.30	28 (20.4)	9 (30.00)	0.25	16 (19.3)	21 (25.0)	0.37
Prior history of cancer, *n* (%)	34 (20.4)	29 (22.7)	5 (12.8)	0.18	26 (19.0)	8 (26.7)	0.34	20 (24.1)	14 (16.7)	0.23
Hypertension, *n* (%)	66 (39.5)	52 (40.6)	14 (35.9)	0.60	54 (39.4)	12 (40.0)	0.95	32 (38.6)	34 (40.5)	0.80
Body mass index (kg/m^2^)	25.7 [22–29.1]	26.2 [22.6–29.4]	24.3 [21.7–27.6]	0.19	25.8 [22.5–29.4]	25.1 [22–27.6]	0.33	23.7 [20.3–27.2]	27.3 [23.3–30.5]	**0.001**
Intensive care unit, *n* (%)	84 (50.3)	56 (43.8)	28 (71.8)	**0.002**	66 (48.2)	18 (60.0)	0.24	0 (0.0)	84 (100.0)	
ARDS, *n* (%)	134 (80.7)	27 (21.26)	100 (78.7)	0.24	105 (77.2)	29 (96.7)	**0.01**	54 (65.9)	80 (95.2)	**< 0.001**
SaO2 (%)	92 [87–96]	93 [88–96]	93 [84–94]	**0.04**	92 [88–96]	92 [84.5–94]	0.25	94 [92–97]	90 [84–93.5]	**< 0.001**
pO2 (mmHg)	64 [55–75]	67 [58–76]	56.5 [50–72.5]	**0.02**	66 [56–76]	61 [49–72]	0.19	68.5 [54–87]	62 [56–74]	0.41
Non-invasive ventilation, *n* (%)	81 (49.7)	56 (45.2)	25 (64.1)	**0.04**	60 (45.1)	21 (70.0)	**0.01**	15 (18.8)	66 (79.5)	**< 0.001**
Duration of non-invasive ventilation (days)	4 [2–7]	4 [2.5–7]	5 [2–8]	0.78	4 [2.5–7]	4 [2–8]	0.48	6 [3–10]	4 [2–7]	0.14
Mechanical ventilation, *n* (%)	40 (24.5)	27 (21.8)	13 (33.3)	0.14	25 (18.8)	15 (50)	**< 0.001**	1 (1.3)	39 (47)	**NA**
Duration of mechanical ventilation (days)	12 [5–19]	11 [6–18]	15 [4–20]	0.83	13 [4.5–18.5]	12 [7–20]	0.75	16	11.5 [5–19]	NA
Venous thrombosis, *n* (%)	39 (23.4)	0 (0.0)	39 (100.0)	**NA**	32 (23.4)	7 (23.3)	0.99	11 (13.3)	28 (33.3)	**0.002**
Pulmonary embolism/thrombosis, *n* (%)	29 (17.4)	0 (0.0)	29 (74.4)	**NA**	23 (16.8)	6 (20)	0.67	3 (3.6)	26 (31.0)	**NA**
**Treatments**										
Corticosteroids, *n* (%)	63 (17.4)	46 (35.9)	17 (43.6)	0.39	50 (36.5)	13 (43.3)	0.48	12 (14.5)	51 (60.7)	**< 0.001**
Antithrombotic therapy, *n* (%)	130 (77.8)	91 (71.1)	39 (100)	**< 0.001**	107 (78.1)	23 (76.7)	0.86	51 (61.5)	79 (94.1)	**< 0.001**
Tocilizumab (anti-IL6), *n* (%)	22 (13.2)	16 (12.5)	6 (15.5)	0.64	18 (13.1)	4 (13.3)	NA	2 (2.4)	20 (23.8)	NA
**Death, *n* (%)**	30 (18.0)	23 (18.0)	7 (18.0)	0.99	0 (0.0)	30 (100.0)	NA	12 (14.5)	18 (21.4)	0.24
**Laboratory findings**										
C reactive protein (mg/L)	159.5 [70–236]	149.5 [52–219.5]	218 [142–296]	**< 0.001**	149.5 [61–237]	177.5 [143–230]	0.22	97 [39–77]	208 [142–284]	**< 0.001**
Hemoglobin (g/dL)	11.6 [9.6–13.3]	11.7 [10.1–13.3]	10.6 [9.1–12.4]	**0.02**	11.65 [9.95–13.3]	10.7 [9.2–12.4]	0.14	11.7 [10.4–13.3]	11.5 [9.5–13.25]	0.26
Platelets (x10^9^/L)	272.5 [193–350]	261 [192–335]	311 [194–404]	0.15	269.5 [194.5–339.5]	290 [185–433.5]	0.32	256 [191–335]	286 [196–379]	0.34
MPV (fL)	10.6 [9.8–11.3]	10.65 [9.85–11.3]	10.5 [9.6–11.2]	0.32	10.6 [9.8–11.3]	10.9 [9.75–11.25]	0.96	10.45 [9.8–11.2]	10.8 [9.85–11.4]	0.17
Leucocytes (x10^9^/L)	8.15 [5.4–10.75]	7.7 [5.3–10.59]	8.5 [6.5–12.3]	0.09	8.0 [5.3–10.4]	9.0 [6.6–12.3]	0.16	7.0 [4.5–8.6]	9.8 [6.8–13.4]	**< 0.001**
Lymphocytes (x10^9^/L)	0.95 [0.59–1.51]	0.98 [0.64–1.54]	0.86 [0.43–1.37]	0.38	0.99 [6.25–1.61]	0.75 [0.25–0.99]	**0.02**	1.05 [0.76–1.67]	0.82 [0.53–1.35]	**0.02**
Neutrophils (x10^9^/L)	6.22 [4.07–8.79]	5.6 [3.75–8.68]	7.23 [8.84–8.86]	0.10	5.81 [3.81–8.69]	7.81 [4.42–10.1]	0.12	4.77 [2.58–6.45]	8.3 [5.6–10.5]	**< 0.001**
Monocytes (x10^9^/L)	0.5 [0.33–0.74]	0.51 [0.34–1.54]	0.48 [0.28–0.71]	0.22	0.52 [0.33–0.75]	0.48 [0.36–0.72]	0.93	0.55 [0.37–0.75]	0.48 [0.32–0.73]	0.30
Neutrophil-lymphocyte ratio	6.39 [3.01–12.96]	5.32 [3.01–12.09]	9.34 [3.11–19.95]	0.06	5.22 [2.82–12.49]	10.39 [6.43–12.96]	**0.023**	3.71 [2.52–6.39]	5.32 [10.89–21.94]	**< 0.001**
Lymphocyte-monocyte ratio	1.93 [1.11–1.89]	1.93 [1.21–2.94]	2.15 [1.04–3.01]	0.85	1.99 [1.24–3.22]	1.5 [0.82–2.14]	**0.013**	1.94 [1.33–3.01]	1.88 [0.92–2.82]	**0.25**
Platelet-lymphocyte count ratio	264.8 [160–414]	248.2 [138.3–385.2]	348.0 [191.3–521.9]	**0.04**	251.5 [160–388.7]	336.8 [189.7–683.9]	0.12	220.5 [135.3–352]	301 [187.6–587.5]	**0.02**
Prothrombin ratio (%)	72 [60–81]	74 [60–82]	68.5 [61–80]	0.55	72 [60–82]	74.5 [59.5–80]	1.00	79.5 [64.5–91.5]	69 [57–80]	**0.02**
aPTT ratio	1.29 [1.11–1.53]	1.29 [1.08–1.5]	1.24 [1.12–1.63]	0.36	1.28 [1.1–1.57]	1.33 [1.17–1.48]	0.53	1.21 [1.0–1.33]	1.36 [1.14–1.65]	**0.007**
D-dimers (μg/L)	1,200 [750–3,214]	1,055 [596–1,962]	1,856 [1,150–8,994]	**0.005**	1,124 [669–3,214]	1,397 [1,127–4,680]	0.15	1,012 [669–1,207]	1,762 [952–6,093]	**0.008**
Fibrinogen (g/L)	6.65 [5.25–7.85]	1.29 [1.08–1.5]	1.24 [1.12–1.63]	0.48	6.6 [5.2–7.9]	6.7 [5.4–7.6]	0.95	5.5 [4.4–6.6]	7.3 [5.9–8.5]	**< 0.001**
IL-6 (pg/mL)	17.7 [6.7–47.9]	14.8 [6.2–45.9]	26.5 [11.8–65.9]	0.14	14.6 [6.0–44.7]	45.2 [16.1–169]	**0.005**	9.4 [3.9–21.4]	33.0 [13.5–104.5]	**< 0.001**
IL-1 (pg/mL)	39.3 [36.1–57.1]	40.5 [36.9–51.6]	37.4 [25.4–61.4]	0.20	39.8 [35.5–60.3]	38.2 [36.5–50.5]	0.88	40.5 [35.7–59.8]	38.2 [36.3–48.9]	0.55
Angiotensin-II (pg/mL)	159 [2.0–782.3]	427 [4.5–797.6]	26.0 [0.5–631.3]	0.21	31.8 [0.9–774.2]	565 [44.5–791.2]	0.21	10.2 [0.5–785.9]	492.9 [15.7–782.3]	0.07
Tissue factor level (pg/mL)	8.0 [4.9–27.0]	8.0 [4.7–24.4]	8.2 [6.0–40.6]	0.47	8.7 [5.5–7.1]	7.1 [3.4–13.7]	0.11	7.2 [5.2–15.0]	11.9 [3.9–38.4]	0.13
Activated monocytes (MFI)	18,592 [12,017–31,408]	19,279 [11,932–31,804]	16,940 [13,286–8,994]	1.00	20,522 [13,189–33,467]	12,561 [8,154–24,221]	0.06	24,707 [15,138–31,804]	16,940 [9,534–36,545]	0.30
Classical monocytes (%)	92.0 [86.1–96.1]	91.8 [84.5–96.0]	94.3 [87.9–97.3]	0.39	92.3 [86.3–96.8]	89.4 [82.1–94.5]	0.10	92.0 [86.2–95.7]	92.0 [85.2–96.2]	0.09
Tissue factor^+^ monocytes (%)	1.74 [0.77–3.84]	1.75 [0.79–4.42]	1.70 [0.66–2.7]	0.37	1.63 [0.75–4.14]	1.76 [0.81–2.57]	0.92	1.37 [0.72–3.27]	2.34 [0.81–3.99]	0.21
Tissue factor HLA-DR^+^ monocytes (MFI)	1,449 [1,041–2,287]	1,461 [1,085–2,393]	1,372 [883–2,105]	0.38	1,504 [1,041–2,450]	1,283 [1,022–1,774]	0.30	1,452 [1,065–3,000]	1,405 [1,016–2,019]	0.30
CD3^+^ T-cells (%)	74.5 [66.9–80.3]	75.1 [68.2–80.3]	71.1 [64.5–81.6]	0.30	74.5 [68.3–80.3]	74.5 [64.3–80.8]	0.57	74.8 [69.3–82.1]	74.5 [66.3–78.4]	0.54
CD4^+^ T-cells (%)	67.7 [58.2–78.0]	66.8 [56.6–76.9]	75.3 [63.3–83.8]	**0.008**	67.4 [58.7–78.0]	69.5 [52.3–80.6]	1.00	61.8 [54.9–73.4]	72.6 [61.4–81.4]	**0.007**
CD8^+^ T-cells (%)	27.5 [17.1–37.4]	28.8 [18.5–39.6]	21.8 [12.2–27.8]	**0.008**	28.3 [17.1–36.9]	24.2 [17.6–41.1]	0.75	31.9 [22.7–40.6]	22.6 [14.8–32.6]	**0.008**
CD4^+^/CD8^+^ T-cell ratio	2.46 [1.59–4.49]	2.31 [1.45–4.06]	3.57 [2.33–6.98]	**0.007**	2.31 [1.45–4.06]	3.57 [2.33–6.98]	0.87	2.37 [1.64–4.49]	2.81 [1.28–4.68]	**0.007**
HLA-DR^+^/CD38^+^/CD4^+^ T-cells (%)	10.7 [7.0–17.8]	11.6 [7–18.0]	6.7 [8.5–16.9]	0.32	10.2 [6.3–16.7]	15.3 [9.9–24.5]	**0.019**	12.5 [8.1–20.8]	9.8 [7.0–16]	0.12
HLA-DR^+^/CD38^+^/CD8^+^ T-cells (%)	34.3 [21.3–50.9]	35.8 [18.3–51.6]	31 [22.1–41.9]	0.45	34.7 [19.9–50.9]	34.0 [28.3–41.7]	0.98	34.4 [25.3–51.6]	34.3 [19.7–47.1]	0.21
ADAMTS13 activity (%)	55 [38.5–77.5]	62 [46–80]	39 [31–68]	**0.002**	66 [41–81]	38.5 [33–50]	**0.004**	73 [47–85]	49 [33–71]	**0.008**
vWF:Ag (IU/dL)	400.5 [312.9–521.5]	383.2 [264.3–522.5]	453.4 [362.4–510]	0.17	385.6 [293.6–509]	463.7 [375.3–529.6]	0.20	293.6 [197.9–410.6]	423.6 [355.7–555.5]	**0.002**
vWF:CB (IU/dL)	351 [282.5–477.5]	346 [236–442]	368 [293–489]	0.17	330 [237–438]	431 [316–546]	**0.03**	285 [181–340]	370 [308–483]	**0.005**
vWF:Ag/ADAMTS13 activity ratio	6.96 [4.22–12.72]	5.56 [3.77–10.76]	5.62 [11.83–19.53]	**0.005**	5.64 [4.10–11.69]	12.26 [7.82–15.45]	**0.02**	4.10 [1.76–5.70]	10.09 [5.32–15.01]	**< 0.001**

VT, venous and pulmonary thrombosis; ICU, intensive care unit; BMI, body mass index; ARDS, acute respiratory distress syndrome; SaO2, oxygen saturation; pO2, oxygen pressure; MPV, mean platelet volume; aPTT, activated prothrombin time; IL, interleukin; TF, tissue factor; MFI, mean fluorescence intensity; VWF, von Willebrand factor. Qualitative variables are reported as numbers and percentages. Quantitative discrete and continuous variables are reported as medians and interquartile ranges (IQR). Pearson’s Chi Square was used for comparison of qualitative variables and Mann-Whitney test to compare quantitative variables between subgroups. Bold values reflect statistically significant values.

### Clinical features associated with VT

**FIGURE 2:**
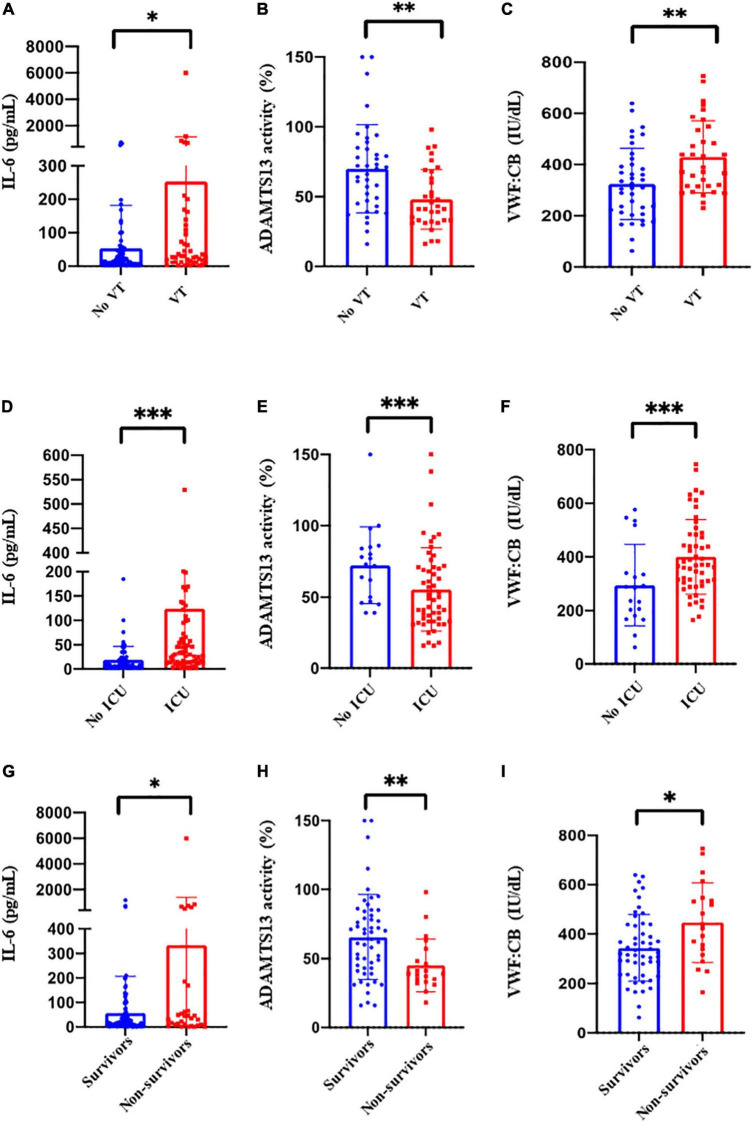
**(A)** C-reactive protein, **(A)** interleukin-6 (IL-6), **(B)** ADAMTS13 activity, **(C)** VWF:CB at the baseline between patients with (red bars) or without (blue bars) venous and pulmonary thrombosis (VT); **(D)** interleukin-6 (IL-6), **(E)** ADAMTS13 activity, **(F)** VWF:Ag, at the baseline between ICU patients (red bars) vs. non ICU patients (blue bars); **(G)** Interleukin-6 (IL-6), **(H)** ADAMTS13 activity, **(I)** VWF:CB at baseline between survivors (blue bars) and non-survivors (red bars). *p < 0.05; **p < <0.01; ***p < 0.001.

Patients with VT were more often admitted in ICU (*p* = 0.002), and had consistently lower O2 saturation (*p* = 0.04), lower pO2 (*p* = 0.02) and more non-invasive ventilation (*p* = 0.04). All these patients received anti-thrombotic therapy. They had more D-dimers (*p* = 0.005), a lower ADAMTS13 activity (*p* = 0.002) with higher vWF:Ag/ADAMTS13 activity ratios (*p* = 0.005), higher CRP levels (*p* < 0.001) and more anemia (*p* = 0.02). CD8+ T-cells (*p* = 0.008) were lower and CD4+ T-cells were higher (*p* = 0.008) in this population (Table 1 and Figure 2).

### Clinical features associated with ICU hospitalization

Patients in ICU differed from those recruited through emergency rooms by several factors. They were younger (*p* < 0.001), more often males (*p* < 0.001) and mostly obese (*p* = 0.001). They presented more often with acute respiratory distress syndrome (ARDS) (*p* < 0.001) and received more non-invasive ventilation (*p* < 0.001). These patients had more VT (*p* = 0.002); they received more corticosteroids (*p* < 0.001) and more anti-thrombotic therapy (*p* < 0.001) (Table 1). They had more inflammation with higher CRP levels (*p* < 0.001), PB polymorphonuclears (*p* < 0.001) and IL-6 (*p* < 0.001), and a higher CD4/CD8 T-cell ratio (*p* = 0.007). Lastly, levels of D-dimers (*p* = 0.008), fibrinogen (*p* < 0.001) and VWF/ADAMS13 ratio (*p* < 0.001) were higher in this population (Figure 2).

### Clinical features associated with survival

Non-survivors were predominantly males (*p* = 0.023) with more ARDS (*p* = 0.01) and requiring more mechanical ventilation (*p* < 0.001); they had more activated CD4+ T-cells (*p* = 0.019), higher IL-6 levels (*p* = 0.005) and a lower lymphocyte count with lower neutrophil-lymphocyte and lymphocyte-monocyte ratios (Table 1). Considering coagulation pathways, patients who deceased had a lower ADAMTS13 activity (*p* = 0.004), higher VWF:CB (*p* = 0.03) and a higher VWF:Ag/ADAMS13 ratio (*p* = 0.02) (Table 1 and Figure 2).

### Predictive features of VT, ICU hospitalization or death at admission

By univariate analysis, CRP level > 150 mg/L, IL-6 ≥ 20 pg/mL, D-dimers > 1,500 μg/L, ADAMTS13 activity ≤ 50%, VWF:Ag ≥ 400 IU/dL, VWF:CB ≥ 350 IU/dL and VWF:Ag/ADAMTS13 activity ratio ≥ 10 were associated with VT, ICU admission or death (Table 2).

**TABLE 2:** Clinical findings on admission associated with VT, death or ICU hospitalization by univariate analysis.

	VT		ICU		Death	
	OR (CI 2.5–97.5%)	*p*	OR (CI 2.5–97.5%)	*p*	OR (CI 2.5–97.5%)	*p*
CRP > 150 mg/L	1.16 (0.51–2.63)	0.73	3.02 (1.01–9.35)	**0.05**	3.19 (1.53- 6.62)	**0.002**
IL-6 ≥ 20 pg/mL	1.51 (0.70–3.32)	0.30	3.96 (1.54–10.15)	**0.004**	3.88 (1.93–7.81)	**< 0.001**
D-dimers > 1,500 μg/L	2.72 (1.00–7.39)	**0.05**	1.28 (0.41–3.99)	0.68	12.6 (2.67–59.59)	**0.001**
ADAMTS13 activity ≤ 50%	3.26 (1.29–8.2)	**0.01**	4.32 (1.49–12.53)	**0.004**	2.09 (0.89–4.91)	0.09
VWF:Ag ≥ 400 IU/dL	1.73 (0.70–4.25)	0.23	2.23 (0.80–6.20)	0.13	1.37 (0.60–3.10)	0.75
VWF:CB ≥ 350 IU/dL	1.25 (0.46–3.42)	0.67	3.39 (1.06–10.88)	**0.04**	1.37 (2.10–21.09)	**0.001**
VWF:Ag/ADAMTS13 activity ≥ 10	2.92 (1.05–8.14)	**0.04**	5.2 (1.60–16.86)	**0.006**	3.89 (1.14–13.28)	**0.03**

VT, venous and/or pulmonary thrombosis; ICU, intensive care unit; OR, odds ratio; CI, confidence interval; CRP, C-reactive protein; TF, tissue factor; MFI, mean fluorescence intensity; VWF, von Willebrand factor. Bold values reflect statistically significant values.

Using these thresholds, a scoring system was built based on the odds ratios derived from significant parameters identified by logistic regression: (i) CRP and VWF (3 points each) and (ii) IL-6 and ADAMS13 activity (4 points each).

The probability to require ICU hospitalization yielded an AUC of 0.74 with a sensitivity and a specificity of, respectively, 67.8 and 63.9% for a score ≥ 4; for a score of ≥ 7 sensitivity was 60.7% and specificity 74.7%. With an AUC of 0.73, death could be predicted with a sensitivity of 76.7% and a specificity of 53.3% for a score of ≥ 4; for a score of ≥ 7 sensitivity was 63.3% and specificity 61.3%. Death was significantly associated with a score of ≥ 4 (*p* = 0.004).

## Discussion

By assessing simultaneously various inflammation and coagulation markers in a large cohort of COVID-19 patients, this study provides a comprehensive description of risk factors for VT, death or hospitalization in ICU in this viral infection. Conducted over 14 months, this work likely involved mostly alpha and beta SARS-CoV-2 variants (25) and provides risk factors associated with these viruses. Here, 23.3% of patients experienced VT. This rate is comparable to this of other non-COVID-19 cohorts with severe infection (26), although the pathophysiological mechanism may differ.

An activated angiotensin-II/IL-6/CRP pathway and a high pro-aggregant VWF/ADAMTS13 ratio, reflecting strong inflammation and endothelial damage axis, appeared as independent risk factors for VT, death and/or ICU hospitalization. This study confirms and extends the view that ADAMTS13 activity is a reliable marker to assess the severity of SARS-CoV-2 infection (10). In this regard, normal levels of ADAMTS13 activity were identified as a strong predictor of survival. By contrast, platelet parameters, previously reported as early predictive markers of severity (27, 28), had no prognostic value in this series (29). Although most prognostic factors identified here confirm previous findings (25), the strength of this work is that multiple markers were addressed simultaneously, allowing to provide a more comprehensive prognostication of patients with COVID-19. These results may therefore help identifying patients for whom a so far undiagnosed thrombosis (especially pulmonary embolism/thrombosis) should be investigated.

The strong association of the IL-6/CRP inflammation pathway with both severe thrombotic events, survival and ICU hospitalization supports therapeutic approaches based on monoclonal antibodies directed against IL-6 alone or with corticosteroids in patients with intermediate severity (30, 31). Interestingly, IL-6 but not CRP levels were significantly discriminative between survivors and non-survivors (27), confirming that IL-6 assessment should be preferred over CRP to evaluate critically ill patients (28). Similarly, the identification of a high pro-aggregant VWF/ADAMTS13 ratio as a strong prognostic factor, opens perspectives for VWF-targeted therapies (13). No influence of TF was observed in this series, confirming previous findings (32), although TF has been reported by others as a potential mediator of pathogenesis in COVID-19 by driving endothelial dysfunction and coagulopathy (29). Moreover, TF-expressing monocytes, that were found to be mildly increased in symptomatic vs. asymptomatic patients (30), were not different here between patient groups; hence, the role of serum or cell surface-expressed TF as a predictive marker of thrombo-embolism in patients with COVID-19 remains debatable.

T cells play are crucial in maintaining immune function and viral clearance. The impact of immune modulation as reflected here by an increased peripheral CD4/CD8 T-cell ratio, was confirmed to segregate patients with VT and those requiring ICU hospitalization. Yet, we did not confirm that a higher peripheral CD4/CD8 T-cell ratio was associated with a better outcome (31, 33), and whether peripheral CD4/CD8 T-cell ratio reflects prognosis in COVID-19 deserves further studies.

Taken together, these observations support a tentative unifying model where SARS-CoV-2 primarily induces a deregulation of the renin-angiotensin-aldosterone system axis. In this model, SARS-CoV-2 S protein binds to and internalizes its receptor ACE2, leading to a lack of conversion of angiotensin-II into the protective peptide angiopoietin_1,7_ and thereby to the accumulation of angiotensin-II (21). In turn, angiotensin-II through the activation of its receptor AT1R induces the production of IL-6 and subsequently CRP. Lastly, the release of IL-6 may at least in part account for the high levels of circulating VWF, but also in the decrease of ADAMTS13 activity, leading to a pro-aggregant phenotype and thrombotic microangiopathy-like features (10, 13).

Altogether, this study suggests that the simultaneous analysis of a combination of multiple key coagulation and inflammatory markers in COVID-19 pathophysiology could refine the prognostication of severe outcome in COVID-19, and allow identifying patients at risk of VT, ICU hospitalization and death, and those in whom a VT (especially a pulmonary thrombosis) should be investigated. The score proposed here, relying on 4 key assessments could be refined/validated by retrospective analyses of COVID-19 patient cohorts where these markers were possibly independently measured. They should also be generalizable to other types of severe viral infections with evidence of prevalent venous and pulmonary thrombosis (34) where their prognostic value could be of help in patient management.

## Data availability statement

The original contributions presented in this study are included in the article/Supplementary material, further inquiries can be directed to the corresponding authors.

## Ethics statement

The studies involving humans were approved by the Ethics committee: CE SRLF 20-29 and CER-Paris-Saclay-2020-050. The studies were conducted in accordance with the local legislation and institutional requirements. The participants provided their written informed consent to participate in this study.

## Author contributions

JZ: Writing – original draft. RB: Writing – original draft. BT: Writing – original draft. GG: Writing – original draft. YC: Writing – original draft. AP: Writing – original draft. NH: Writing – original draft. MR: Writing – original draft. BJ-L: Writing – original draft. AV: Writing – original draft. CC: Writing – original draft. PC: Writing – original draft.

## Footnotes

1
https://www.r-project.org/
