# Supplementary material for: Combined coagulation and inflammation markers as predictors of venous thrombo-embolism and death in COVID-19
Source: Front Med (Lausanne). 2024 Jun 10;11:1399335. doi: 10.3389/fmed.2024.1399335 (PMC11194426; doi:10.3389/fmed.2024.1399335)
Supplement: Supplementary file 1 [file Table_1.DOCX]

|  | **fluorochrome** | **clone** |
| --- | --- | --- |
| CD45 | V500 | HI30 |
| CD3 | Percp-Cy5.5 | SK7 |
| CD4 | APC | RPA-T4 |
| CD8 | FITC | SK1 |
| CD14 | PE-Cy7 | M5E2 |
| CD16 | V450 | 3G8 |
| CD38 | APC-R700 | HIT2 |
| HLA-DR | APC-Cy7 | L243 |
| CD142 | PE | HTF-1 |

**Supplemental Table 1** : Antibodies used for flow cytometry experiments. All from BD Biosciences
